# Using Oral Microbiota Data to Design a Short Sucrose Intake Index

**DOI:** 10.3390/nu13051400

**Published:** 2021-04-21

**Authors:** Anders Esberg, Linda Eriksson, Pamela Hasslöf, Simon Haworth, Pernilla Lif Holgerson, Ingegerd Johansson

**Affiliations:** 1 Department of Odontology/Cariology, Umeå University, SE 901 87 Umeå, Sweden; linda.eriksson@umu.se (L.E.); ingegerd.johansson@umu.se (I.J.); 2 Department of Odontology/Pedodontics, Umeå University, SE 901 87 Umeå, Sweden; pamela.hasslof@umu.se (P.H.); pernilla.lif@umu.se (P.L.H.); 3 Medical Research Council Integrative Epidemiology Unit, Department of Population Health Sciences, Bristol Medical School, University of Bristol, Bristol BS8 2BN, UK; simon.haworth@bristol.ac.uk; 4 Bristol Dental School, University of Bristol, Bristol BS1 2LY, UK

**Keywords:** sucrose, sucrose index, saliva microbiota, caries

## Abstract

Excessive sucrose consumption is associated with numerous health problems, including dental caries, and is considered to play a critical role in shaping the human microbiota. Here, we aimed to confirm the association between sucrose exposure and oral microbiota profile, develop a short food-based index capturing variation among sucrose consumers and validate it against oral microbiota and dental caries in a derivation cohort with 16- to 79-year-old participants (*n* = 427). Intake and food preferences were recorded by questionnaires and saliva microbiota by 16S rDNA sequencing. Taxonomic similarities clustered participants into five clusters, where one stood out with highest sucrose intake and predicted sugar related metabolic pathways but lowest species diversity in the microbiota. Multivariate modelling of food intake and preferences revealed foods suitable for a sucrose index. This, similarly to sucrose intake, was related to bacterial pattern and caries status. The validity of the sucrose index was replicated in the population-based Gene-Lifestyle Interactions in Dental Endpoints (GLIDE, *n* = 105,520 Swedish adults) cohort. This suggested that the index captured clinically relevant variation in sucrose intake and that FFQ derived information may be suitable for screening of sucrose intake in the clinic and epidemiological studies, although adjustments to local consumption habits are needed.

## 1. Introduction

A growing body of research aims to understand the role of gastro-intestinal microbiota in health and disease and the determinants of niche specific communities [1]. Multiple studies imply that dysbiosis of the gastro-intestinal microbiota is associated with diseases including inflammatory bowel diseases, obesity, type 1 and type 2 diabetes, autism/ADHD, and certain gastrointestinal cancers [2]. Dysbiosis in the oral cavity may be related to oral cancers, rheumatoid arthritis, dental caries and periodontitis [3,4,5,6]. Diet is suggested to affect both the oral and gut microbiotas, e.g., fermentable carbohydrates, primarily sucrose, for the oral microbiota [7] and meat, fibers and sugars for the gut microbiota [2]. If so, then dietary modification to regulate the microbiota may be an important public health strategy given the high global prevalence of overweight, obesity and type 2 diabetes [8]. Studies have therefore searched for associations between sugar intake, mainly added sugar or sugar-sweetened beverages, and total mortality and non-communicable diseases (NCD) [9,10]. Though data support a role for sugar in such diseases, dental caries is the only disease to date with experimental substantiation for a causal role of sugar in disease aetiology.

Most national guidelines recommend that energy from sucrose should not exceed 10% of total energy intake [11,12] and WHO even suggests 5% as a target [13]. Monitoring sucrose intake or other aspects of diet is labor intense and challenging due to underreporting. Attempts have been made to find a biomarker for sucrose or sugar intake in blood, urine, saliva or tooth biofilms but without success [14]. Accordingly, assessment of sugar exposure relies on self-reported intake. Thus, labor-effective and validated measures of sugar exposure would be valuable in clinical settings and in large scale epidemiological studies.

Given the role of sucrose for dental caries development and related microbiota [15] we hypothesized that oral microbiota data could be used to empirically design a short dietary index which captures biologically relevant dietary variation in sucrose index. Thus, the aims of the present study were to (i) evaluate associations between lifestyle related factors and oral microbiota to confirm association between sucrose exposure and oral microbiota profile in the general population, (ii) derive a short sucrose intake index to classify subjects by sucrose intake and (iii) validate the index by testing for association with oral microbiota and dental caries in the derivation cohort and with dental caries in a replication group.

## 2. Materials and Methods

### 2.1. Study Cohort

The derivation cohort was recruited from (a) young health participants (16–21 years old; *n* = 217) as they came for an annual dental examination at a dental clinic in Umeå, Sweden, and (b) adults living in the northern part of Sweden who volunteered to participate upon request (21–79 years old; *n* = 210). The exclusion criteria were cognitive disability, severe illness, recent antibiotic treatment and inability to communicate in Swedish or English.

### 2.2. Recording of Food Intake and Food Preferences

The participants in the derivation cohort were asked to complete two electronic food questionnaires. The first questionnaire was a semi-quantitative food frequency questionnaire (FFQ) which asked about habitual intake over the latest 12 months. The FFQ included 93 food items/food aggregates which was an expansion with nine additional questions of an 84-item validated FFQ [16]. The questions were designed to capture the Swedish food panorama, and the nine added questions were on energy drinks, one on sugar substitute sweetened products and seven non-sugar containing foods common in vegetarian diets. A total of 15 of the FFQ questions represented foods with added sucrose, five questions foods with intrinsic sugar (sucrose and monosaccharides, i.e., juice, berries and fruits), and one question non-sugar sweetened products. Intake frequencies were recorded on an increasing nine-level scale. The options were never, less than once a month, 1–3 times per month, once a week, 2–3 times a week, 4–6 times a week, once a day, 2–3 times a day and four or more times a day. Portion sizes for staple foods, meat/fish and vegetables were estimated from four photographs. Other portions were based on natural portions, such as an egg, from portion indications given at The National Food Administration (www.livsmedelsverket.se, accessed on November 2020). Reported intakes were transformed to intakes per day and daily intake of energy, sucrose and other nutrients were calculated by weighting according to information given at The National Food Administration.

In the second questionnaire the participants indicated their preference for foods representing sour, bitter, sweet or neutral taste, including preference for eight foods representing sweet taste. The likings were indicated on a six-level scale (“love”, “like”, “it is ok”, “not so good”, “dislike”, “hate” (with an additional option “do not know” [17].

### 2.3. Recording of Lifestyle Factors

Information on education level, general health status, current medications, oral hygiene habits, bleeding gums, tobacco use (smoking and use of Swedish snus (snuff) and alcohol intake was collected in a questionnaire connected to the FFQ).

### 2.4. Microbiota Analysis

Stimulated saliva was collected for 3 min into ice-chilled sterile test tubes while participants chewed on a 1 g piece of paraffin wax. Collection was performed at least 3 h after nearest meal or tooth brushing and samples were stored at −80 °C until used.

Bacteria 16S rDNA amplicons were generated from the v3-v4 regions by PCR on DNA extracted from saliva, a mock community and a negative control (ultra-pure water) using fusion primers with 341F (ACGGGAGGCAGCAG) forward and 806R (GGACTACHVGGGTWTCTAAT) reverse primers as described by Caporaso [18]. Equimolar 16S rDNA amplicon libraries were pooled and purified using AMPure XP beads (Beckman Coulter, Stockholm, Sweden) and sequenced using the Illumina Miseq platform. The samples were spiked with 5% PhiX (Illumina, Stockholm, Sweden) and two mock samples and two negative controls were included in each run.

Obtained sequences were de-multiplexed, pair-end reads fused, primers, ambiguous, chimeric and PhiX sequences removed, and amplicon sequence variants (ASVs) retained using the open-source software package DADA2 in the QIIME2 next-generation microbiome bioinformatics platform (https://qiime2.org accessed on November 2020) [19,20]. ASVs were taxonomically classified against the expanded Human Oral Microbiome Database (eHOMD) (http://www.homd.org accessed on November 2020) [20,21]. ASVs with at least two reads and 98.5% identity with a named species or unnamed phylotype in eHOMD were retained, and those with the same Human Microbial Taxon (HMT) ID number were aggregated. The HMT aggregated taxa were standardized to the level of the sample with fewest reads (19,700 reads), and then transformed by inverse hyperbolic sine transformation. Inverse hyperbolic sine transformation defines log values, including for zero-values, which are prevalent for bacterial species.

For simplicity, all named species and unnamed phylotypes are referred to as species in the text.

### 2.5. Prediction of Oral Microbiota Functions from the 16S rRNA Gene Sequences

The molecular functions of the oral microbiota were predicted from the obtained 16S rRNA sequences using Phylogenetic Investigation of Communities by Reconstruction of Unobserved States (PICRUSt2) [22] and the KO Database of Molecular Functions by ortholog annotation (KEGG orthologues, KO, https://www.genome.jp/kegg/ko.html accessed on November 2020) within QIIME2. The steps included creating a closed reference feature table using the Greengenes database version 13_5 (http://greengenes.lbl.gov accessed on November 2020) which is trained against PICRUSt2, estimation of diversity core-metrics in QIIME2, and export of a KEGG KO feature table for downstream analyses.

### 2.6. Replication Cohort

Replication of a potential sucrose index for total sucrose intake was tested in an independent cohort with data on dietary intake, caries status and potential confounders in adults. This cohort (GLIDE; Gene-Lifestyle Interactions in Dental Endpoints) includes dental data from 1999 to 2015 obtained from dental electronic records in the county of Västerbotten, Northern Sweden and linked diet and lifestyle screening data from the Västerbotten Intervention Programme (VIP). VIP is a population-based program where inhabitants in the county of Västerbotten, Sweden, are invited to a health screening with a medical examination and recording of diet, lifestyle and living conditions. The participation rate has varied over time, with an average of 60% [23]. Selection bias, as regards to income, socio-economic group, employment, education, smoking and medical measures, has been found marginal [24]. The cohort has been described previously [25].

In the replication cohort participants completed an FFQ which between 1985 and 1996 included the first 84 questions in the derivation cohort FFQ. Of the 15 questions about products with added sucrose in the derivation cohort FFQ, 14 questions were the same (the lacking question was for intake of energy drinks). From 1996 the FFQ was reduced to 66 questions by merging some foods with similar nutrient profiles and excluding some food that were rarely consumed. In the shorter version 13 questions on products with added sucrose remained unaltered and the questions on sweets and sugar lumps/granulated sugar were merged but with the identical text retained.

### 2.7. Caries Scoring

Caries status was recorded by one dentist in the 16–21-year-old participants in the derivation cohort and linked from electronic dental records in the replication cohort. In the younger group tooth surfaces (S) with decay in the enamel or extending into the dentine (De), with a filling (F), or were missing (M) were recorded from visual and radiographic examinations. No teeth were lost due to caries in this group and caries was expressed as the total number of decayed and filled tooth surfaces (DeFSs). For the participants in the replication cohort information on caries was linked from electronic records kept at the Public Dental Clinics in the catchment area. Caries examinations were done as described for the discovery cohort apart from that the decay component was restricted to cavities extending into the dentine (D) to reduce examiner variation (DMFS score) [25].

### 2.8. Ethical Approval

The basic study was approved by the Swedish Ethical Review Authority (dnr 2018/335-31 and dnr 09-134M), and the GLIDE replication cohort by the Regional Ethical Board at Umeå University (later transformed to the Swedish Ethical Review Authority (dnr 09-134M with an addendum in 2015) and by the Swedish Data Inspection Board (dnr 471-2011). The study followed the Helsinki declaration including that all participants signed informed consent to participate.

### 2.9. Data Handling and Statistical Analysis

Participants with >10% of the FFQ answers or a portion size missing were excluded. Participants with implausible estimated energy intake (food intake level (FIL, energy intake divided by basal metabolic rate [26]) below the 1st or above the 99th percentile, height <30 cm or >210 cm, body weight <35 kg, BMI <15.0 kg/m^2^ or missing were excluded as described earlier [27]. Nutrient intakes were energy adjusted to 2000 kCal for women and 2500 kCal for men but also evaluated as energy provided in proportion of total energy intake (E%) and residuals from regression on energy intake. Tertile and quintile classifications were done in sex and 10-year age strata.

Agglomerative hierarchical clustering (Ward method) was used to classify participants with similar microbiota profiles, and diversity measures were compared by PERMANOVA using Paleontological Statistics (PAST4) [28], with 9999 permutations to correct *p*-values for false discovery rate.

Descriptive estimates and testing, i.e., means with standard deviation (SD) or 95% confidence limits (CI), proportions (%), univariate non-parametric testing, and ranking classification by sex and age group were done using SPSS version 25 (IBM Corporation, Armonk, NY, USA). For group comparisons, BMI was adjusted for sex and age using general linear modelling. Nutrient intakes were also adjusted for BMI and energy intake and caries scores for BMI. For microbiota comparisons, Shannon and Simpson alpha diversity measures were calculated using QIIME2. All tests were two-sided and *p*-values <0.05 were considered statistically significant unless false discovery rate correction at FDR 0.05 was applied to account for multiple testing [29].

Multivariate analyses included unsupervised principal component analysis (PCA) for group separation and partial least square regression discrimination analysis (PLS-DA) to compare selected groups. PLS analysis was also used to identify foods and food preferences associated with estimated daily sucrose intake (g/day). Here SIMCA P+ version 15.0 (Sartrius Stedim Data Analytics AB, Malmö, Sweden) was used and variables were scaled to unit variance and a K-fold cross-validation was performed by systematic removal of every 7th observation and prediction of the remaining observations (Q^2^-values). Results were displayed in score loading plots (2-dimensional projection with the maximal separation of the observations) or a column loading plot representing PLS correlation coefficients with 95% CI. Coefficients where the 95% CI did not include zero were considered statistically significant and variable in projection (VIP) values >1.5 as highly influential in the model.

## 3. Results

### 3.1. Derivation Cohort and Oral Microbiota Characteristics

The derivation cohort comprised 427 participants between 16 and 79 years of age, with 61.6% females and 59.5% below 25 years (Table 1). Mean (SD) BMI was 23.1 (3.2) with 24.3% having a BMI ≥ 25 (overweight or obese), 6.4% were current or past smokers and 12.9% were current or former Swedish snus (snuff) users (Table 1). Estimated daily intake of carbohydrates was 217 g, protein 83 g, fat 106 g and sucrose 31 g (Table 1).

In total, salivary DNA was available for 416 participants, and 16S rDNA sequencing yielded 21,517,630 sequences after quality filtering and chimera removal. These represented 9473 ASVs of which 5211 ASVs matched to a sequence in eHOMD at ≥98.5% identity. These represented 116 genera and 465 species or phylotypes with two or more reads (Tables S1 and S2). The negative controls yielded (mean (SD) 141 (52) reads and the species in the mock communities were identified.

Cluster classification of the participants by genera abundances yielded five clusters with the best conceptual fit and were compared. These cluster groups were assumed to, besides the microbiota phenotypical similarity, represented similarities in diet or other lifestyle characteristics. Age, proportion overweight, sucrose intake, daily tooth brushing and gum bleeding on tooth brushing differed significantly between the cluster groups (Table 1). In summary, cluster 4 stood out for a seemingly poorer lifestyle and cluster 2, with fewest overweight participants and fewest with gum bleeding and the lowest sucrose intake, seemed to have the healthiest lifestyle. Cluster 5 only harbored young (<25 years old) participants.

**Figure 1:**
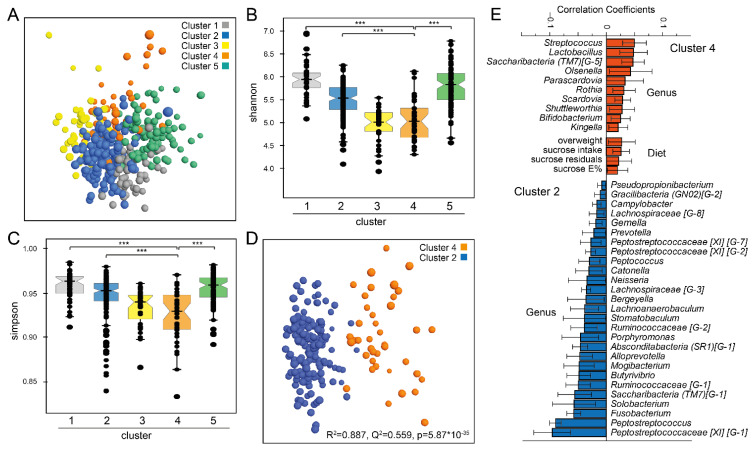
(**A**) PCA score plot based on the relative abundances of the 116 genera, and Box-jitter plots of (**B**) Shannon and (**C**) Simpson diversity indices. (**D**) PLS-DA score plot of participants in cluster 2 and 4 with (**E**) PLS correlation coefficients with 95% CI of genera and sucrose measures (x-variables) that were significantly influential for the separation of the two clusters. Color coding follows that indicated in (**A**) throughout the figure. Group differences were evaluated by PERMANOVA, *** *p* < 0.001 (FDR adjusted).

PCA multivariate modelling from the abundances of the 116 genera confirmed separation of the participants in the five clusters. Though the separation was not fully displayed in the 2-dimensional score plot (Figure 1A), alpha diversity differed significantly between the five clusters with the lowest diversity in cluster 4 with the highest sucrose intake (Figure 1B,C). The separation of cluster 4 and cluster 2 with the highest and lowest sucrose intake, respectively, became evident in PLS-DA modelling (Figure 1D). Genera that were significantly influential for being in cluster 4 versus 2, included *Streptococcus*, *Lactobacillus*, *Saccharibacteria* (*TM7*) [G−1], *Olsenella, Parascardovia, Rothia, Scardovia* and more (Figure 1E). Several species in the genera seen in Figure 1E contributed to the higher abundances in cluster 4, such as *Streptococcus mutans*, *Streptococcus gordonii*, *Streptococcus anginosus, Streptococcus intermedius, Scardovia wiggsiae* and several lactobacilli. For some genera, species detection was split among clusters, i.e., *Saccharibacteria* (*TM7*) (Figure 1D, Table S3).

### 3.2. Predicted Function of the Species Profile in Cluster 4 Versus 2

**Figure 2:**
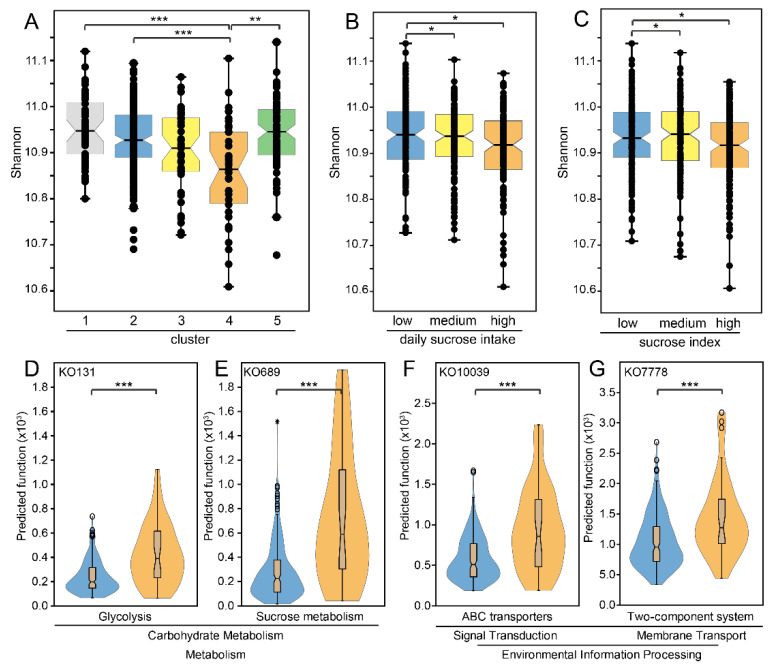
Comparisons of predicted microbiota function (KEGG orthologues, KO) based on 16S rRNA gene sequence information in PICRUSt2. BOX-jitter plots based on Shannon diversity of the predicted functions in (**A**) the 5 clusters, and tertile groups of (**B**) estimated daily sucrose intake and (**C**) a created sucrose index. (**D**–**G**) Violin plots illustrating two functions linked to carbohydrate metabolism [(**D**) glycolysis and (**E**) sucrose metabolism], and two functions linked to environmental information processing [(**F**) ABC-transporter and (**G**) two-component system]. Group differences were evaluated by PERMANOVA, * for *p* < 0.05, ** *p* < 0.01, and *** *p* < 0.001 (FDR adjusted).

The predicted functional diversity based on pathways from orthologue annotation (KEGG orthologues, KOs) of the ASV sequences was lowest in microbiota-based cluster 4 with the highest estimated sucrose intake (Figure 2A) and in the top tertile group from sucrose intake distribution (Figure 2B). Top 200 functions enriched in cluster 4 compared with cluster 2 were identified by PLS analysis and correlation coefficients (Table S3). Of these 200 functions, 19 were not significant in univariate analysis after correction for multiple testing (FDR, 0.05%, *p* < 0.0007) and 12 functions were poorly characterized. Among the remaining 169 functions enriched in cluster 4, 80 associated with metabolism. i.e., carbohydrate metabolism (*n* = 20), glycan biosynthesis and metabolism (*n* = 16), and amino acid metabolism (*n* = 10). Environmental information processing was the second largest functional family (*n* = 49), with three major subgroups, i.e., membrane transport (*n* = 21), signalling and cellular processes (*n* = 17) and signal transduction (*n* = 9) linked to e.g., ABC-transporter (*n* = 12) and two-component system (*n* = 9).

### 3.3. Discovery of a Short Index for Sucrose

Univariate analyses found significantly higher energy adjusted sucrose intake in cluster 4 than cluster 2 (Table 1), which, along with optional approaches to account for potential diet underreporting, e.g., percent of total energy intake (sucrose E%) or as residuals from regression on estimated energy, was supported in the PLS-DA regression (Figure 1E). This, combined with higher predicted functions related to sucrose metabolism, supported an association between sucrose intake and overall microbiota profile in vivo. This was potentially further supported by a numerically higher, though not statistically significant, caries prevalence among 16–21-year-old participants (*n* = 217, 83.4% with a sign of manifest or initial caries) in cluster 4 than 2 (mean (95% CI): 7.2 (3.6, 10.8) versus 3.5 (1.7, 5.4), *p* = 0.079).

**Figure 3:**
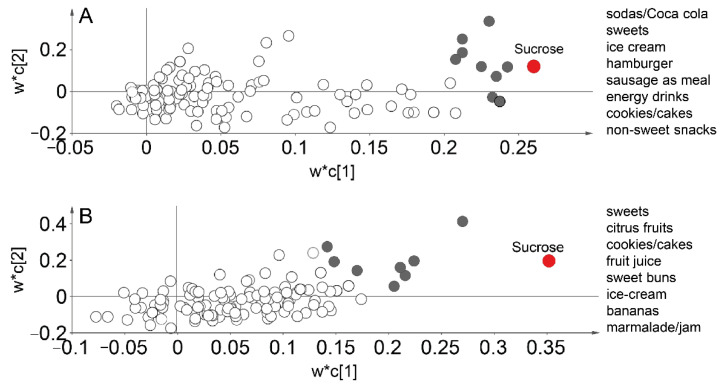
Loading plots from PLS regression to identify food intake and preferences associated with estimated daily sucrose intake (g/day). The upper figure (**A**) refers to the younger group and the lower figure (**B**) to the older group. Red dots represent the dependent variable and grey dots foods significantly influential for the variation of the dependent variable. Unfilled circles indicate non-influential food intakes and preferences. The models were strong with an explanatory power (R^2^) of 0.92 and 0.87 and a cross-validated predictive power (Q^2^) of 0.78 and 0.62 for the two groups (**A**,**B**), respectively. w*c refers to the X loading weight (w), and Y loading weight (c) combined to one vector showing the relationships between X and Y, for component [1] and component [2].

To identify possible markers for inclusion in a short sugar intake index, multivariate PLS regression was undertaken with estimated sucrose intake as the dependent variable and food intakes from the 93 FFQ-questions and scores for food preferences as the response cloud. This was modelled separately for those <25 and ≥25 years to account for inter-generational differences in diet patterns. Daily intakes of sodas/Coca cola, sweets, ice cream, hamburger, sausage as meals, energy drinks, cookies/cakes, sweet buns and non-sweet snacks (e.g., crisps and popcorn) were identified as the strongest markers (i.e., a statistically significant PLS correlation coefficient ≥0.1 and a VIP-value >1.5) in the younger group (Figure 3A). In the older group, daily intake of sweets, citrus fruits, cookies/cakes, fruit juices, sweet buns, ice cream, bananas, and marmalade/jam were the strongest markers for total sucrose intake (Figure 3B). No food preference was associated with estimated sucrose intake in the young group, and in the older group, only a preference for sweet buns was significantly associated with the outcome.

**Figure 4:**
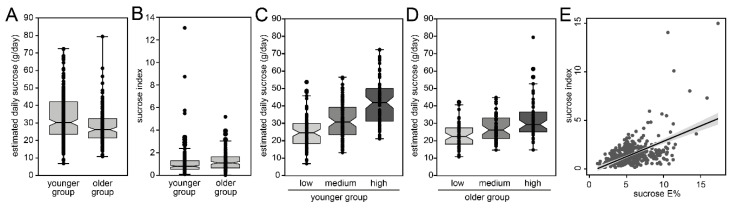
Box plots illustrating the distributions of (**A**) estimated daily sucrose (g/day) and (**B**) the sucrose index by age group, (**C**,**D**) estimated sucrose intake in tertile groups (low, medium, high) based on the distribution of the sucrose index in the (**C**) younger and (**D**) older group, and (**E**) scatter plot between sucrose index values and sucrose E% values with regression line (95% CI).

An index for estimated daily sucrose intake (“sucrose index”) was calculated as the sum of reported intake frequencies for the foods identified by PLS. The index included 9 food items in the younger group and eight food items in the older group from the 93 questions in the full FFQ. The distribution of estimated sucrose intake and the sucrose index values in the younger and older age groups, respectively, are shown in Figure 4A,B. The mean (95% CI) of the sucrose index was 1.47 (1.17, 1.77) and 1.64 (1.48, 1.80), for the younger and older age groups respectively, and for both age groups daily intake of sucrose increased with increasing sucrose index (tertile groups, *p*-values for trend <0.001, Figure 4C,D). Partial correlation coefficients (controlling for sex and age) between the sucrose index on the one hand and estimated daily sucrose intake, sucrose E% or sucrose residuals on the other were between 0.70 and 0.60 (all *p* < 0.001), whereas protein and total fat intakes were weakly negatively correlated (−0.30 and −0.29, *p* < 0.001). Additional controlling for BMI did not affect the associations.

Linear regression of estimated sucrose index values on sucrose E% values revealed a beta-value of 0.41 in the study group with similar results for the two age groups, i.e., 0.48 and 0.35, respectively (Figure 4E). Thus, a sucrose index value of ≤3 would correspond to the recommended maximum intake of 10 E% from sucrose.

**Figure 5:**
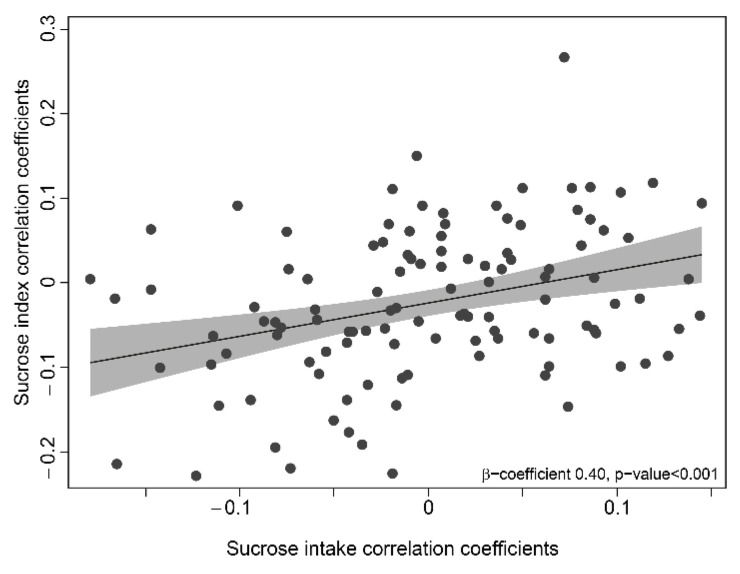
Scatter plot of the coefficients from Spearman correlations between the abundances of the 116 genera and sucrose intake and the sucrose score, respectively, and regression line with 95% CI.

The sucrose index, for which the creation was triggered by the finding of an association between saliva microbiota and sucrose intake, was validated against individual microbiota profiles. First, the correlation pattern between sucrose intake and abundances of the 116 genera compared with that between the sucrose index values and the same abundances showed a significant trend for the respective rankings (*p* < 0.001; Figure 5).

**Figure 6:**
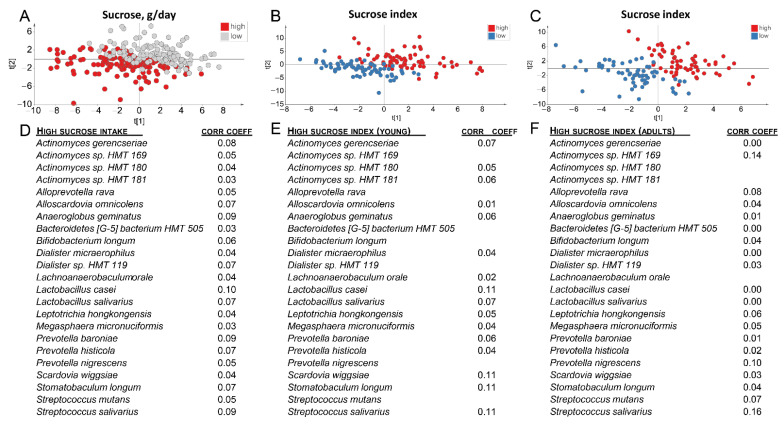
(**A**–**C**) PCA scatter plots showing separation of participants in the highest versus lowest tertile from (**A**) reported daily sucrose intake among all participants, and (**B**) sucrose index distributions in the younger group and (**C**) sucrose index distributions in the older group. (**D**) Bacterial species with a significant impact in the separation seen in (**A**), and (**E**,**F**) in the separation seen in (**B**,**C**), respectively. The models included abundances of the 465 saliva identified species and phylotypes as the independent variable block (X), and the independent variables in the PLS models were tertile classification of reported daily sucrose intake and sucrose index, respectively. Species lacking a correlation coefficient were not influential in the model.

Second, PLS modelling with abundances of the 465 identified species largely separated participants in the highest versus lowest tertile based on daily sucrose intake (g/day) in the score plot (Figure 6A). The same independent block (465 bacterial species abundancies) also separated participants in the highest versus lowest tertile based on the sucrose index scores (Figure 6B,C). Species that were significantly influential for the separation in Figure 6A, i.e., column loading correlations coefficient where the 95% CI did not cover zero, are listed in Figure 6D and most of these were also influential in the separation based on the sucrose index (Figure 6E,F).

### 3.4. Evaluation of the Sucrose Index in an Independent Adult Replication Cohort

**Figure 7:**
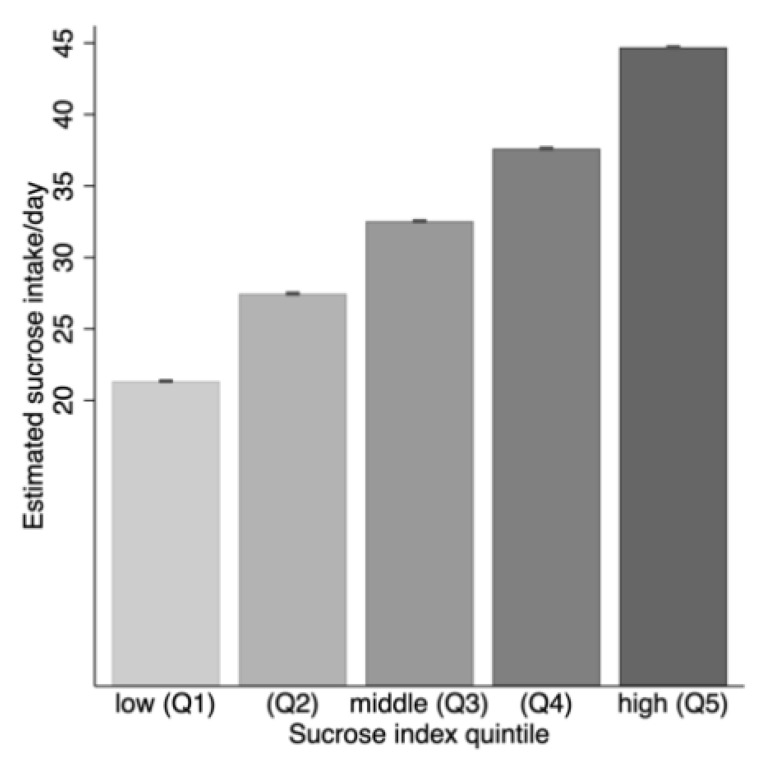
Bar graph illustrating mean (SD) estimated daily sucrose intake (g/day) by quintile groups based on the sucrose index value distribution in the replication cohort.

The sucrose index algorithm for the older group in the derivation cohort was applied to FFQ information from the independent, population-based replication cohort (*n* = 105,520; 19–71 years; 51.0% women). Mean (SD) BMI in the replication cohort was 26.0 (4.3) and 53.8% had a BMI ≥ 25, and estimated mean (SD) sucrose intake was 34.0 (16.8) g/day (Table 2). The derived mean (SD) sucrose index values was (2.0 (1.3) in the entire cohort and higher among women than men (2.0 (1.2) and (1.9 (1.3), respectively. Similar to in the discovery cohort, mean sucrose intake increased by increasing sucrose index values (Figure 7).

### 3.5. Sucrose Score and Caries

**Figure 8:**
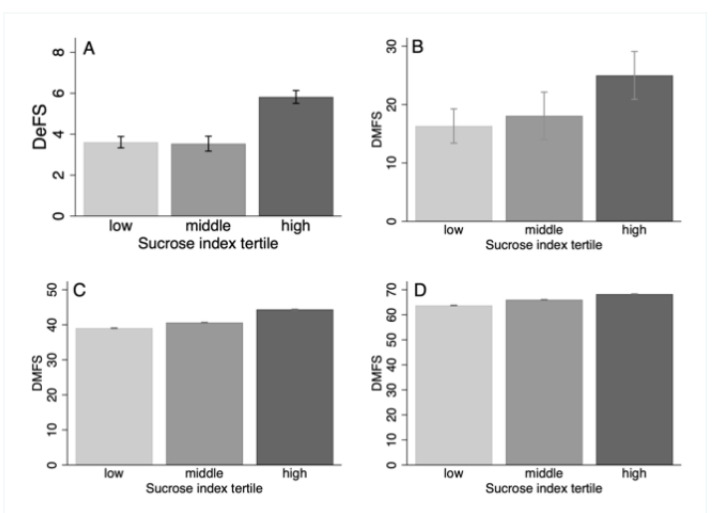
Bar graphs showing sex, age and BMI adjusted mean with 95% CI for caries in (**A**) the 16–21-year-olds (*n* = 217) in the derivation group, and (**B**) 30–33-year-olds in the replication cohort (*n* = 119), (**C**) 38–44-year-olds in the replication cohort (*n* = 10,523), and (**D**) 55–64-year-olds in the replication cohort (*n* = 10,518) by tertile classification from the sucrose index distribution in the replication cohort.

Young participants in the derivation group with the highest sucrose index had the highest caries prevalence (mean DeFS (SD) 5.9 (1.3)) compared to those in the middle 3.5 (1.5) and lowest 3.6 (1.2) tertiles (*p*_trend_ < 0.001) (Figure 8A). Similarly, the highest prevalence (mean DMFS) was seen in the tertile with the highest sucrose index in all 3 age groups in the replication cohort. The strongest difference was seen in the youngest 10-year age groups, and, though remaining statistically significant, the difference flattened by increasing age group (Figure 8B–D).

## 4. Discussion

In the present study, we aimed to derive a short index for dietary sucrose intake and validate this index by testing for association with sugar-related microbiota traits and dental caries experience as a model for a sugar-related disease outcome. Initially, we characterized saliva microbiota by 16S rDNA amplicon sequencing in 416 participants between 16 and 79 years. After clustering the participants by bacterial taxonomic similarities, one group was defined by the lowest species diversity but highest predicted sugar related metabolic pathways in their saliva microbiota and with the highest estimated sucrose intake. This suggested the oral microbiota as a possible trait suitable for at least high sucrose intake identification. Following this, we evaluated reported intake of 93 foods/food clusters and selected a short index based on eight or nine markers. In multivariate PLS modelling, this short intake index predicted 87 and 62% of the variation in estimated sucrose intake in the younger and older groups, respectively, in the derivation cohort. The relative validity of the sucrose index was supported by similar association pattern between detected microbiota traits and estimated sucrose intake and the sucrose index, respectively. In both the derivation cohort and in a large independent population-based cohort the short index was associated with caries experience, suggesting the index captures clinically relevant variation in dietary risk factors for disease. We therefore suggest the index may be suitable for convenient classifying persons by sucrose intake in the clinic and large-scale studies, although we note possible need for adjustment of index foods depending on local consumption habits.

The present finding of aggregation of the participants into five distinct groups based on similarities in the saliva microbiota and with deviating sucrose intake levels confirms results from our previous study in a smaller study group [15] and is in accordance with reduced diversity reported from experimental studies [30] and amplicon sequencing studies in children [31], which does not necessarily mean lower overall bacterial abundances. To our knowledge this is, however, the first study to also demonstrate sucrose associated altered metabolic functions based on prediction from amplicon sequences in microbiota-based cluster groups. Altered gene regulation and expression have been shown in experimental studies using single or a few species, including the cariogenic *S. mutans* [32]. In line with such experimental studies, the present study found *S. mutans* and other saccharolytic and aciduric species, such as *S. wiggsiae* [33] and species in *Bifidobacterium* and *Lactobacillus* [15] to characterize the cluster group with highest sucrose intake. In concert, these findings support a role of sucrose as a determinant of a caries associated microbiota profile and the predominant ecological plaque hypothesis explaining the establishment of caries-related dysbiotic tooth biofilm community [34]. Recently, sugar has also been suggested to be associated with compositional traits of fecal microbiota [35].

Despite experimental evidence for causality, epidemiological studies in recent decades have struggled to demonstrate association between sucrose intake and caries. This has several possible explanations, such as high intake with limited variation, enhanced disease resistance at the population level due to e.g., fluoride treatments, or difficulties in accurate assessment of sugar intake [36]. This highlights the need for a valid biomarker or other convenient way to estimate sucrose intake. To date, no validated biomarker has been described [14], though promising results are reported for specific metabolic-based sugar markers in urine and plasma [37,38,39].

While these methods are promising, urine and blood are not readily utilized in the clinical settings, such as dental clinics, and sample collection may be demanding in sizable observational studies. Estimates of sugar exposure, often combined with that of fat, or sugar containing soft drinks have been targeted in shorter FFQs but these are built on 25 or more questions [40]. The present sucrose index, which was based on 8 or 9 foods reflecting sucrose containing foods per se but also other lifestyle reflecting foods, captured most of the variation in estimated sucrose intake which is captured by much longer questionnaires, and was associated with sugar-related microbiota phenotypes in the discovery cohort. It was also associated with caries prevalence in an independent validation cohort and may therefore be useful for classification of sucrose intake in clinical settings or observational studies. Through its short format it is also suitable for an app-development for patient feedback.

Recent studies have suggested that a person’s preference for sweet taste is associated with higher sugar intake, whereas a preference for bitter intake relate to lower sugar intake [41,42], which we have confirmed in the population where the present participants were recruited [15,17]. It has also been suggested that scores from a taste preference-based instrument may be used to estimate sweet intake in children [43] but this could not be confirmed for a combined score or single foods in the present adult derivation cohort. Interestingly, citrus fruits and fruit juice were among the stronger markers for having higher sucrose intake. This was an unexpected finding and it cannot be ruled out that this is a cohort effect but it may be noteworthy that we in a previous publication [17] found a genetic correlation between sweet and sour taste traits for the *TAS1R1*, *TAS1R2* and *GNAT3* genes, possibly indicating shared pathways for sweet and sour taste.

Using linear regression, a sucrose score of ≤3 was found to correspond to the generally recommended intake of no more than 10% of the total energy intake to come from sucrose [14]. The score could therefore be useful in counselling as a convenient way to identify patients who may be exceeding current guidelines. We caution however that any clinical application must be taken in the context of other risk factors, for example in dental caries, which is a multifactorial disease, with risk factors including genetic susceptibility [44], undernutrition [45], systemic disease and medication [46] as well as sucrose intake. The effect of these additional risk factors, many of which increase with age, may explain why the association between sucrose intake and caries prevalence was weaker in the older age groups. Defining appropriate levels between caries risk and sucrose intake may be delineated for groups of patients based on refined disease measures, such as suggested in a recent study in Swedish twins [47], and possibly in combination with complex multiplex omics-analyses, such as genomics, epigenomics, metagenomics, metabolomics and proteomics [48].

The main strengths of this study are the use of biological data from microbiota profiling and disease experience to validate the proposed index. Nevertheless, there are limitations in the study that should be considered in the interpretation, comparisons with other studies, and suitability for application in populations with different food habit profiles. The main limitation relates to the inevitable bias in self-reported diet intake, and especially underreporting of sugar intake [49]. Therefore, it is a weakness that the “gold standard” method, here FFQ estimated sucrose intake, and the source of foods for the sucrose index are affected by similar systematic errors. This weakness is not unique to the present study but occurs for any “gold standard” method based on self-reported intake [50] and is inevitable given the current lack of biological biomarkers for sucrose intake. The present study was limited to sucrose and did not include assessment of other simple sugars or high fructose corn syrup. From an oral microbiota point of view both sucrose and monosaccharides lead to low pH but sucrose induces additional metabolic and functional effects on the oral bacteria and biofilms [51]. One advantage of the proposed index is that information on portion size and composition are not required, potentially reducing errors caused by portions size estimates and selection of marker foods and dishes used for weighting from a large number of similar foods and dishes in national databases. Though we suggested that the overall strategy for the construction of the sucrose index is applicable in any population, local amendments and purpose of use need to account for differences in dietary patterns and supply.

## 5. Conclusions

The conclusions from the present study are that, in line with experimental studies, the saliva microbiota profile reflects sucrose intake in the general population. A food-based index constructed as a proxy for sucrose exposure may serve as a convenient tool in clinical use or large-scale studies where a full record of the diet intake is not feasible. This is under the assumption that the foods building the index are relevant for the intended population. Further confirmations of the validity under different conditions are also recommended.

## Figures and Tables

**Table 1:** Characteristics of the participants in the derivation cohort.

Characteristics	All Study	Clusters Groups					
	Participants	C1	C2	C3	C4	C5	*p*_groups_
Number ^1^	427 ^1^	54	179	45	40	98	
Sex, % females	61.6	61.1	64.2	71.1	45.0	60.2	0.136
Age, years, mean (SD)	29.6 (15.9)	31.7 (17.9)	33.1 (16.9)	31.5 (14.6)	32.4 (17.2)	18.6 (1.0)	<0.001
BMI ^2^, kg/m^2^, mean (SD)	23.1 (3.2)	23.1 (3.5)	22.7 (3.0)	23.2 (2.7)	23.8 (3.5)	23.7 (3.4)	0.081
Overweight, % with BMI ≥ 25	24.3	26.9	19.2	26.2	42.5	24.0	0.042
Current or past smoker, %	6.4	7.7	6.5	2.4	10.0	6.2	0.588
Current or past snuff user, %	12.9	15.4	9.4	16.7	17.5	13.6	0.732
Education ^3^, % highest level for age	46.6	45.1	53.0	50.0	35.9	46.7	0.378
Diabetes in close family, %	13.3	11.5	18.2	19.0	10.3	2.1	0.003
Physically inactive, %	33.4	46.2	28.1	26.2	32.5	39.6	0.071
Tooth brushing daily, %	85.9	82.7	97.6	100.0	85.0	76.8	<0.001
Gum bleeding on brushing, %	18.7	26.9	11.8	21.4	27.5	25.0	0.034
Protein ^2^, g/day, mean (SD)	83 (19)	81 (21)	83 (17)	84 (24)	79 (16)	83 (21)	0.517
Fat ^2^, g/day, mean (SD)	106 (22)	109 (22)	108 (23)	103 (21)	106 (23)	102 (20)	0.063
Carbohydrates ^2^, g/day, mean (SD)	217 (40)	213 (38)	213 (41)	215 (33)	224 (43)	223 (40)	0.175
Sucrose ^2^, g/day, mean (SD)	31 (12)	31.1 (14)	29.7 (13)	30.2 (9)	36.9 (14)	31.3 (11)	0.028

^1^ 11 participants lacked microbiota information and other numbers may vary slightly due to single missing data; ^2^ BMI was adjusted for sex and age and dietary measures also for estimated energy intake and BMI; ^3^ Highest level for age refer to upper secondary school or university as appropriate for age.

**Table 2:** Characteristics of the participants in the replication cohort.

Characteristics	Replication Cohort
Number ^1^	105,520
Screening period	1991–2016
Sex, % females	51.0
Age, years (mean (SD))	46.6 (9.0)
BMI ^2^, kg/m^2^ (mean (SD))	26.0 (4.3)
Overweight, % with BMI ≥ 25	53.8
University, %	29.9
Diabetes in close family, %	20.0
Physically inactive, %	64.1
Protein ^2^, g/day (mean (SD))	83.1 (15.1)
Fat ^2^, g/day (mean (SD))	88.5 (22.2)
Carbohydrates ^2^, g/day (mean (SD))	263.2 (45.6)
Sucrose ^2^, g/day (mean (SD))	34.0 (16.8)

^1^ The numbers vary slightly for some measures due to single missing data; ^2^ BMI was adjusted for sex and age and dietary measures also for BMI.

## Data Availability

The data that support the findings of this study are available from the corresponding author upon reasonable request.

## Supplementary Materials

The following are available online at https://www.mdpi.com/article/10.3390/nu13051400/s1, Table S1: Mean (95% CI) for the abundances of genera identified among all participants and in cluster groups, Table S2: Mean (95% CI) for the abundances of species/phylotypes identified among all participants and in cluster groups, Table S3: Predicted orthologue functional annotations based on the 16S rDNA sequences.
